# Local field potentials in the BNST in patients with OCD: acute effects of DBS after symptom provocation

**DOI:** 10.1192/j.eurpsy.2022.1907

**Published:** 2022-09-01

**Authors:** C. Bervoets, H. Heylen, B. Nuttin, M. Mc Laughlin

**Affiliations:** 1 UPCKULEUVEN, Adult Psychiatry, leuven, Belgium; 2 KULEUVEN, Neurosciences, leuven, Belgium; 3 KULEUVEN, Exp Orl, leuven, Belgium

**Keywords:** Deep brain stimulation, obsessive-compulsive disorder, local field potential, symptom provocation task

## Abstract

**Introduction:**

Obsessive-compulsive disorder (OCD) is a disabling psychiatric disorder that affects 2-3% of the population. Pharmacological or cognitive behavioral therapy can reduce symptoms. Deep brain stimulation is emerging for treatment-resistant patients.

**Objectives:**

We measured neuronal activity in two patients with treatment-resistant OCD, who had DBS electrodes implanted bilaterally in the BNST. Local field potentials were recorded directly from the BNST during and without symptom provocation and without electrical stimulation.

**Methods:**

In two patients with a diagnosis of treatment resistant OCD (TR-OCD) local field potentials (LFP) were recorded as part of their clinical follow up post-implantation. In both patients, the diagnosis of TR-OCD was confirmed by a neuropsychiatric examination and a multidisciplinary committee comprising both experienced psychiatrists and neurosurgeons from different medical centers in Belgium. We used BrainSense recording technology in the Percept PC to record the LFPs. The LFP recordings in the first patient were acquired on the 15th day after DBS surgery. In the second patient, the interval between implantation and recording was 18 days. Symptom provocation was performed using the MOCCS image set, developed by Mataix-Cols.

**Results:**

At rest, relative power peaks in the BNST were highest in the theta (4-8 Hz) frequency band for both patients. In both patients switching DBS ON during provocation images appears to cause the LFP signal to closely resemble that recorded during neutral images.

**Conclusions:**

The main finding of this pilot study is that switching stimulation ON in the BNST during provocation images causes the LFP signal to more closely resemble the LFP recorded during neutral images.

**Disclosure:**

No significant relationships.

